# Fabrication and Characterization of Electrospun Keratin Mats with *Echinacea purpurea* L. and Biosynthesized Silver Nanoparticles

**DOI:** 10.3390/ijms26209919

**Published:** 2025-10-12

**Authors:** Akvilė Andziukevičiūtė-Jankūnienė, Erika Adomavičiūtė, Carmen Gaidau, Virgilijus Valeika, Aistė Balčiūnaitienė, Jonas Viškelis, Maria Rapa, Virginija Jankauskaitė

**Affiliations:** 1 Department of Production Engineering, Kaunas University of Technology, 51424 Kaunas, Lithuania; akvile.andziukeviciute-jankuniene@ktu.edu (A.A.-J.); erika.adomaviciute@ktu.lt (E.A.); 2 The National Research & Development Institute for Textiles and Leather-Division Leather and Footwear Research Institute, 031251 Bucharest, Romania; carmen_gaidau@hotmail.com; 3 Department of Polymer Chemistry and Technology, Kaunas University of Technology, 51424 Kaunas, Lithuania; virgilijus.valeika@ktu.lt; 4 Lithuanian Research Centre for Agriculture and Forestry, Institute of Horticulture, 54333 Babtai, Lithuania; aiste.balciunaitiene@lammc.lt (A.B.); jonas.viskelis@lammc.lt (J.V.); 5 Faculty of Materials Science and Engineering, National University of Science and Technology Politehnica Bucharest, 060042 Bucharest, Romania; maria.rapa@upb.ro

**Keywords:** *Echinacea purpurea* L., silver nanoparticles, green synthesis, electrospinning, wool keratin micro/nanofibers

## Abstract

This study presents the development of antibacterial electrospun nanofibrous mats composed of keratin and polyethylene oxide, incorporating *Echinacea purpurea* L. (*EchP*) and green-synthesized silver nanoparticles (*bio*AgNPs) produced using *EchP* extract. The successful synthesis of *bio*AgNPs was confirmed by colorimetric analysis, FTIR, XRD, and TEM. In vitro assays demonstrated antibacterial activity against both Gram-positive and Gram-negative bacteria at ~0.6 µg/mL. Keratin, extracted from sheep wool, retained partial native structure, supporting biocompatibility and cellular regeneration. Incorporation of *EchP* or *bio*AgNPs reduced solution viscosity by 25–45%, significantly affecting mat morphology and shifting fiber diameters toward the 50–100 nm range. Quantitative phytochemical analysis, conducted via UV-Vis spectrophotometry, showed 2–3 times higher release of tannins and phenolic compounds compared to hydroxycinnamic acid derivatives and flavonoids. Keratin electrospun mats with *bio*AgNPs exhibited about 1.5-fold lower polyphenol release, confirming the dual role of polyphenols as electron donors in Ag^+^ bioreduction and as stabilizers.

## 1. Introduction

Biopolymers are a diverse class of macromolecules derived from various renewable sources, including plants (e.g., polysaccharides such as cellulose, starch, and alginate), microorganisms (e.g., polyesters like poly(3-hydroxyalkanoate) derivatives), and animals (e.g., proteins such as collagen, keratin, gelatin, zein, and casein; and polysaccharides like chitosan and hyaluronate) [1]. The primary advantages of biopolymers include: (i) their potential to serve as sustainable alternatives to petroleum-based polymers, and (ii) their biodegradability, biocompatibility, and low toxicity, which make them highly suitable for biomedical applications [2,3].

Keratin is a structural fibrous protein abundantly present in animal-derived materials such as feathers, wool, hair, nails, and horns. It is characterized by a high cysteine content, which enables the formation of disulfide bonds that provide mechanical strength and structural stability. Owing to its biodegradability, biocompatibility, self-assembling ability, and abundance as an agricultural by-product, keratin has emerged as a promising raw material for the development of sustainable biomaterials [4,5]. These properties make keratin particularly suitable for use in wound healing, tissue engineering, drug delivery, and regenerative medicine [6,7,8,9] Since keratin-based materials can be processed into various forms, including films, fibers, hydrogels, scaffolds, and nanoparticles, enabling their application not only in the biomedical field but also across multiple sectors such as textile and cosmetic industries, composites and structural materials, and the packaging industry [4,10]. Recently, keratin has gained attention as a biopolymer for sustainable packaging. Keratin-based films and coatings, often blended with other biopolymers or plasticizers, exhibit biodegradability, antimicrobial activity, and barrier properties, making them suitable for food packaging and single-use items [11].

Electrospinning has become a key technique for fabricating keratin-based nanofibers due to its ability to produce materials with high surface area, porosity, and structural similarity to the extracellular matrix (ECM) [12]. However, pure keratin exhibits poor mechanical properties and low electrospinnability, primarily due to its low molecular weight (9–60 kDa) and limited chain entanglement [4]. To overcome these limitations, keratin commonly is blended with synthetic or natural polymers such as polyethylene oxide (PEO) [13], polyvinyl alcohol [14], chitosan [15], and other polymers [7,16], which improve solution viscosity, fiber formation, and mechanical strength. Recent studies have shown that keratin-polymer composite nanofibers not only enhance the mechanical integrity of the mats but also retain the bioactivity of keratin, promoting cell infiltration and uniform fiber morphology [4]. These keratin-based composites can be further functionalized with bioactive agents [17], drugs [18], metal nanoparticles [19,20], etc.

Silver nanoparticles (AgNPs) have attracted considerable attention due to their unique physicochemical properties, including high surface area, electrical conductivity, chemical stability, and potent antimicrobial activity [21]. Traditionally, AgNPs are synthesized via chemical or physical methods, which offer precise control over particle size and morphology. However, these methods often involve toxic reagents, high energy consumption, and environmental hazards, limiting their suitability for biomedical applications. In contrast, green synthesis of nanoparticles has emerged as a sustainable and eco-friendly alternative. This approach utilizes biological entities, particularly plant extracts, as reducing and stabilizing agents. Phytochemicals such as flavonoids, polyphenols, terpenoids, and alkaloids facilitate the bioreduction of silver ions (Ag^+^) to metallic silver (Ag^0^), while simultaneously stabilizing the nanoparticles [22]. The resulting AgNPs are typically biocompatible, less cytotoxic, and suitable for medical and environmental applications. The plant-mediated synthesis of AgNPs offers additional advantages, such as cost-effectiveness, scalability, and reduced synthesis time. Studies have shown that AgNPs synthesized from various plant parts (leaves, stems, roots, bark) exhibit size ranges between 10–50 nm, with tunable surface plasmon resonance and enhanced rigidity [23].

*Echinacea purpurea* L. is a well-known medicinal plant with a long history of use in traditional medicine, particularly for its immunomodulatory, antioxidant, antibacterial, and anti-inflammatory properties. Its rich phytochemical composition includes caffeic acid derivatives (e.g., chicoric acid, caftaric acid, caffeic acid), alkylamides, flavonoids, polysaccharides, and glycoproteins [24]. These compounds contain functional groups (e.g., hydroxyl, carboxyl, and amide) capable of donating electrons, thereby facilitating the reduction of silver ions (Ag^+^) to elemental silver (Ag^0^) and stabilizing the resulting nanoparticles to prevent aggregation [25]. In particular, chicoric and caffeic acids have been highlighted for their strong antioxidant properties and electron-donating capacity, making them especially effective in nanoparticle synthesis [26].

The findings indicated that keratin electrospinning, especially in combination with other polymers and bioactive additives, offers a versatile platform for developing biocompatible, biodegradable, and functional nanofibrous materials. Therefore, the aim of this study is to develop keratin/PEO-based nanofibrous mats enriched with natural bioactive agents from *Echinacea purpurea* L. (*EchP*) and biosynthesized silver nanoparticles (*bio*AgNPs) in order to create biocompatible and functional materials with antibacterial and bioactive properties for advanced applications.

## 2. Results and Discussion

### 2.1. Keratin Hydrolysate Synthesis and Characterization

The basic physical and chemical characteristics of wool keratin hydrolysate to be synthesized are listed in Table 1, and its molecular weight analysis data are presented in Table 2 and Figure S1 (in Supplementary File).

**Figure 1:**
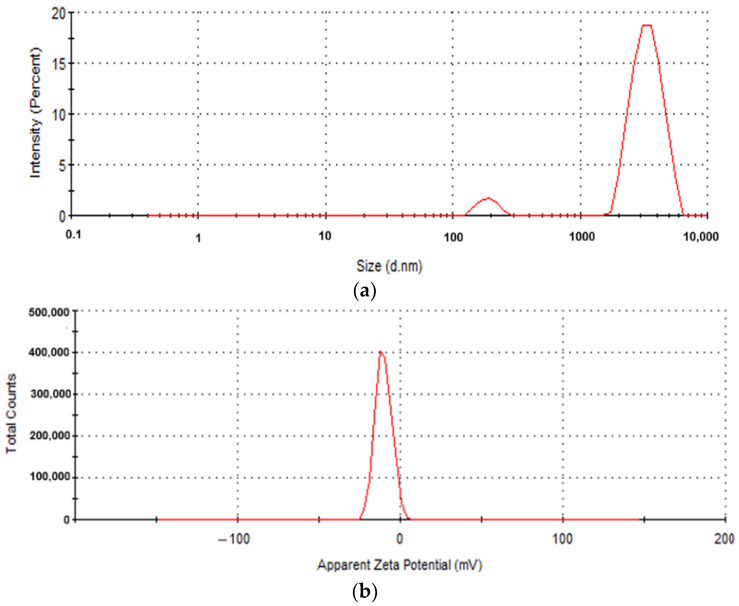
Keratin hydrolysate particle size distribution by intensity (**a**); Zeta potential of −11.4 mV (**b**).

The keratin used has an average molecular weight ranging from 11,315 Da to 14,000 Da (89.2%), a high polydispersity (0.42 according to DLS and 1 according to GPC) and exhibits associative properties. Large molecules (10.8%) and average particle size of 2353 nm were detected using SDS-PAGE electrophoregram analysis. Dynamic light scattering (DLS) analysis identified two populations of keratin hydrolysate particles with sizes of 3441 nm and 188.9 nm (Figure 1).

**Figure 2:**
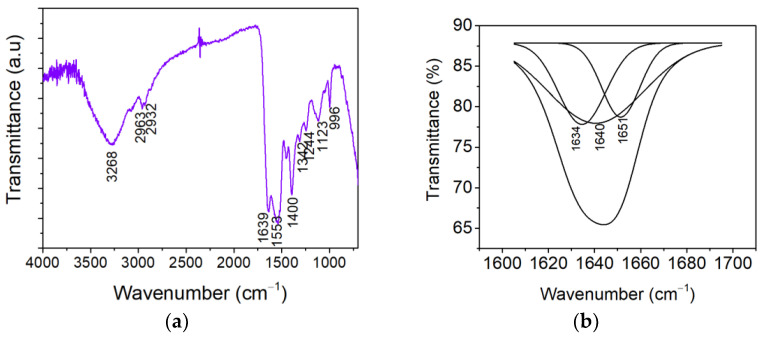
(**a**) ATR–FTIR spectrum of keratin hydrolysate powder; (**b**) Deconvolution spectra of amid I band of keratin hydrolysate.

Figure 2a shows the ATR-FTIR spectrum conducted for keratin hydrolysate, which is characterized by the presence of a wide band around 3268 cm^−1^, corresponding to the stretching vibration of N–H groups (Amide A band). The band at 1639 cm^−1^ can be attributed to the stretching vibration of C=O groups (Amide I band), and 1553 cm^−1^ indicates N–H in-plane bending vibrations characteristic of β-sheet conformation (Amide II band) [27,28,29]. A peak observed at 2932 cm^−1^ can be attributed to the v(CH_3_) in the keratin sample. Moreover, this peak has been interpreted as having a dual effect of Fermi resonance, resulting from the interaction between the overtone of the v(CH_3_) mode in the methyl groups at the ends of the acyl chains. Peaks at 2963–2932 cm^−1^ (–CH_2_–), 1639 cm^−1^ due to the stretching vibration of C=O groups (Amide I band), and 1553 cm^−1^ due to the N–H in-plane bending vibrations for β-sheet conformation (Amide II band) were also observed. The band at 1342 cm^−1^ is attributed to the bending deformation of the CH_3_ in the amino acid, while the bands around 1244 cm^−1^ can be associated with the β-sheet structure of Amide III [27,30]. The ionic character of keratin becomes apparent at 1400 cm^−1^ and the absorption band located at 996 cm^−1^ is associated with the C–SH group. The vibration of cySO_2_–S–Cy group within the keratin structure is indicated by the presence of the peak at 1123 cm^−1^ [28].

FTIR characterization was performed to identify the secondary structure of proteins by deconvolution of the amide I region (1700–1600 cm^−1^) of keratin hydrolysate (Figure 2b). It can be seen that the proteins exhibit different combinations of secondary structures. In Table 3, the shares of β-sheets, α-helix, and random conformations of keratin hydrolyzate are compared to the wool composition. In keratin hydrolysate, the proportion of α-helix structures decreased, and the proportion of β-sheets increased. It is known that the transition of α-helix to β-sheets is assigned to the unfolding of molecules and the breaking of hydrogen bonds [28]. The results suggest that the keratin hydrolyzate obtained from wool still preserves part of the native structure of keratin, which ensures the premises of good biocompatibility and biostimulation of cellular regeneration [31].

### 2.2. Morphology and Properties of EchP Extract and bioAgNPs

**Figure 3:**
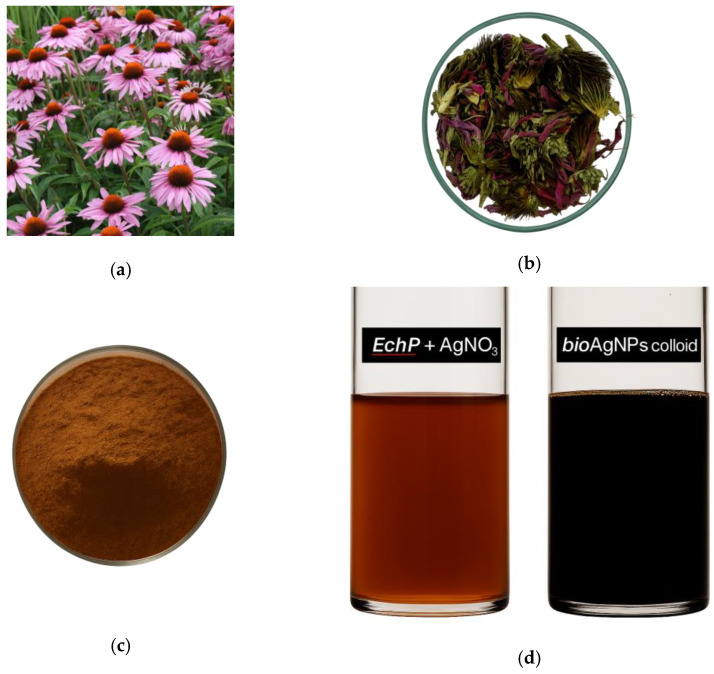
Raw materials used for the preparation of *Echinacea purpurea* L. (*EchP*) extract: fresh flowers (**a**), dried flowers (**b**), ground powder (**c**), and color change observed in the colloidal solution during biosynthesis of silver nanoparticles (*bio*AgNPs) (**d**).

The studies of *EchP* have discovered a large range of bioactive compounds, such as flavonoids, alkaloids, tannins, furochromones, glycosides, polyacetylenes, etc. [33]. Fierascu et al. [34], along with other researchers [25], reported that *EchP* can be successfully used for the green synthesis of biologically active AgNPs. In the present study, the formation of *bio*AgNPs was visually confirmed by a distinct color change in the reaction mixture. Initially, the *EchP* extract mixed with silver nitrate (AgNO_3_ ) exhibited a brownish-orange hue. Upon successful reduction of Ag^+^ ions and nanoparticle formation, the solution transitioned to a dark ruby red, indicating the presence of colloidal *bio*AgNPs (Figure 3d). 

The progression of *bio*AgNP formation in the *EchP* aqueous extract was monitored through changes in CIELAB colorimetric parameters, as summarized in Table 4. Color measurements were expressed using three coordinates: L* (lightness), which indicates the luminance of the color; a*, representing the green–red axis; and b*, representing the blue–yellow axis. From these primary coordinates, two derived parameters were calculated: chroma (C*), indicating color saturation, and hue angle (*h*), representing the color tone. A gradual darkening of the solution, reflected by a decrease in the L* value, suggests a reduction in light scattering, likely due to an increasing concentration and aggregation of *bio*AgNPs. Concomitant decreases in a* and b* values indicate a shift in chromaticity, with the solution becoming less saturated and shifting away from the red-yellow quadrant of the color space. This is further supported by a marked decline in chroma (C*) from 9.76 to 4.88, and a reduction in hue angle (*h*) from 43.62° to 31.63°, indicating a perceptible change in the color tone of the extract over time.

It is well established that *EchP* contains a variety of hydroxycinnamic acids, including caffeic and ferulic acids, as well as their derivatives such as chlorogenic, caftaric, and chicoric acids, which contribute significantly to its biological activity [35]. In this study, UV-Vis spectrophotometric analysis was employed to evaluate the total content of key phytochemical groups—namely proanthocyanidins, hydroxycinnamic acid derivatives, total phenolic content (TPC), and total flavonoid content (TFC)—in both the extract of *EchP* and the colloidal solution of *bio*AgNPs. As shown in Table 5, the concentration of these phytochemicals was markedly reduced in the *bio*AgNP solution compared to the native *EchP* extract. Specifically, the biosynthesis process, initiated by the addition of AgNO_3_ to the plant extract, resulted in a 38% reduction in proanthocyanidins and a 62% reduction in hydroxycinnamic acid derivatives. The TFC also decreased substantially (by 44%), while the reduction in TPC was comparatively moderate (19%). These reductions can be attributed to the role of polyphenolic compounds and flavonoids as both reducing and stabilizing agents during the synthesis of *bio*AgNPs. By donating electrons to Ag^+^ ions, these phytochemicals facilitate the reduction process and contribute to the stabilization of the resulting nanoparticles, thereby depleting their concentration in the final colloidal solution [36,37].

The observed depletion of phytochemicals in the colloidal solution of *bio*AgNPs compared to the native *EchP* extract is consistent with earlier reports on plant-mediated AgNP synthesis. Multiple studies have shown that phenolic compounds and flavonoids act as primary reducing and stabilizing agents, resulting in their significant consumption during nanoparticle formation. For example, Pradeep et al. demonstrated a marked reduction in phenolic acids and flavonoids after AgNP biosynthesis with *Hypericum perforatum* extract, attributing this to their electron-donating and capping roles [38]. Similarly, reviews on green synthesis using various plant extracts report substantial decreases in phytochemical content post-synthesis, with the extent of reduction depending on the initial concentration and reactivity of the compounds [39].

**Figure 4:**
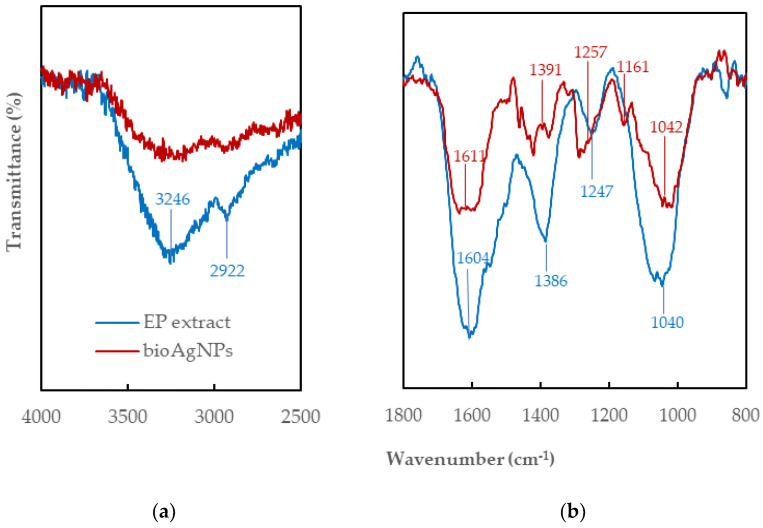
FTIR spectra for *EchP* and *bio*AgNPs: (**a**) 4000–2500 cm^−1^ region; (**b**) 1800–800 cm^−1^ region.

FTIR was used to identify functional groups in *EchP* extract that could be liable in the Ag^+^ ion reduction, nanoparticles stabilization and capping (Figure 4). In the spectrum a symmetric and asymmetric stretching of O–H group and H-bonded stretching in polyphenolic compounds and alcohols can be seen in the region of 3440–3200 cm^−1^ [40]. The region of 3020–2850 cm^−1^ indicates –CH, –CH_2_ and –CH_3_ stretching vibrations derived from carbohydrates in plant extract (Figure 4a). The stretching vibration of the carbonyl group (C=O) causes a strong and distinctive absorption at 1741 cm^−1^ [41]. The peak around 1645 cm^−1^ is associated with amide I (C–O stretching and C–N bending) [42]. The peaks between 1600 and 650 cm^−1^ represent the carbohydrate fingerprints. Those at 1593 cm^−1^ and 1492 cm^−1^ correspond to C=C stretching vibrations from aromatic rings. The 1421 cm^−1^ and 1458 cm^−1^ bands appear due to the combination of O–H and C–H bending, and CH_2_ scissoring in monosaccharides, respectively [43]. Strong peak at 1369 cm^−1^ corresponds to the phenolic O–H groups bending in-plane [44], while the absorbance band at 1280 cm^−1^ is attributed to the stretching vibration of –CO group of polyols, which are intensively involved in the reduction of Ag^+^ ions [45]. Several peaks in the range 1180–1051 cm^−1^ are associated with the stretching vibrations of C–O from alcohol, carboxylic acid, ester and ether. C–H bending is characteristic in the range of 675–1000 cm^−1^ (Figure 4b).

The Ag^+^ ions produced by the addition of aqueous AgNO_3_ were reduced by the functional groups of plant secondary metabolites. Plausible mechanism of silver ions bioreduction to metallic silver nanoparticles presented in [25,36]. Similar peaks are found in the FTIR spectrum of *bio*AgNPs as for *EchP*. In this case peaks at 3310, 3195, 2960, 1741, 1645, 1508, 1438, 1369, 1221 cm^−1^ show the presence of C–H stretching, O–H, C–H bending, C=O, C–N, C=C ring, C–H, phenolic O–H, C–O, respectively. In the FTIR spectrum of *bio*AgNPs, the observed decrease in the intensity of some absorption bands, their shift or disappearance confirm the participation of hydroxyl, carbonyl, and amine groups in the bioreduction and capping process [25,36,46].

**Figure 5:**
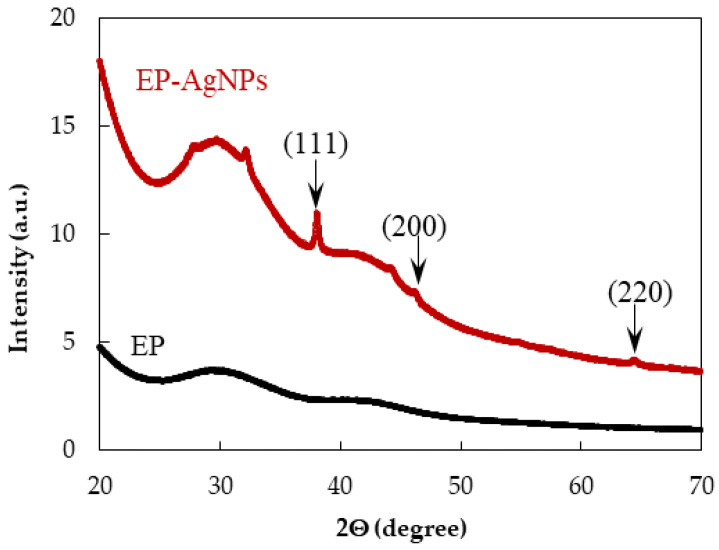
XRD curves of *EchP* and *bio*AgNPs.

XRD data of *EchP* extract and *bio*AgNPs are depicted in Figure 5. The control thin film of plant extract exhibits no distinct diffraction peaks, indicating its predominantly amorphous nature. In contrast, the XRD pattern of the synthesized *bio*AgNPs displays well-defined diffraction peaks at 2θ values of 38.1°, 44.4°, and 64.6°, which correspond to the (111), (200), and (220) crystallographic planes, respectively, of the face-centered cubic (fcc) structure of metallic silver (JCPDS, No. 04-0783). Additionally, a broad shoulder peak observed around 2*θ* = 30° can be attributed to the presence of amorphous organic compounds from *EchP* absorbed onto the surface of the *bio*AgNPs.

**Figure 6:**
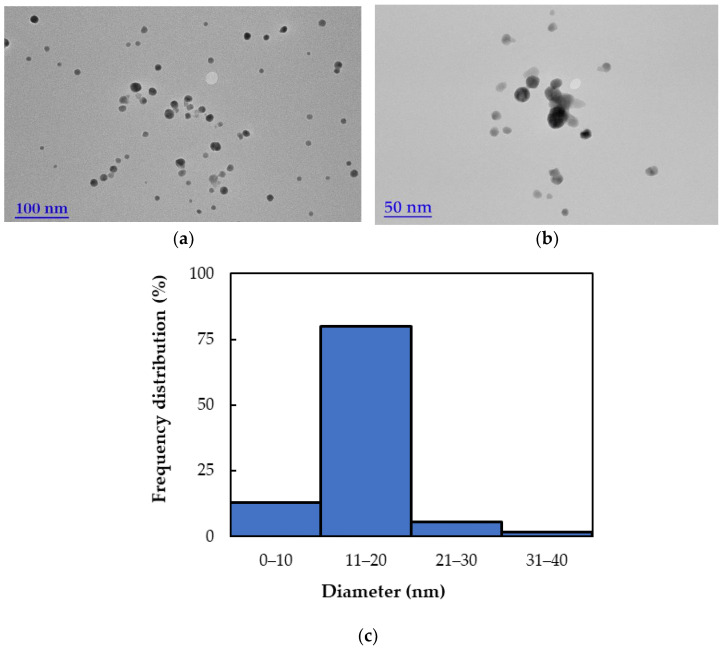
(**a**,**b**) TEM images of *bio*AgNPs at different magnification; (**c**) Frequency distribution of nanoparticle diameters.

Based on the analysis of the TEM micrograph (Figure 6), the *bio*AgNPs are predominantly spherical in shape and exhibit high polydispersity, indicating a wide range of particle sizes. However, the distribution is skewed toward smaller sizes, with the majority (approx. 80%) of particles falling within the 11–30 nm range and an average diameter of 20.5 nm. This morphological diversity is characteristic of green-synthesized nanoparticles, where plant-derived biomolecules influence nucleation and growth processes, resulting in varied particle sizes [36]. Additionally, the nanoparticles appear relatively well-dispersed with minimal agglomeration, suggesting effective stabilization—likely due to the capping action of *EchP* biomolecules involved in the synthesis process.

**Figure 7:**
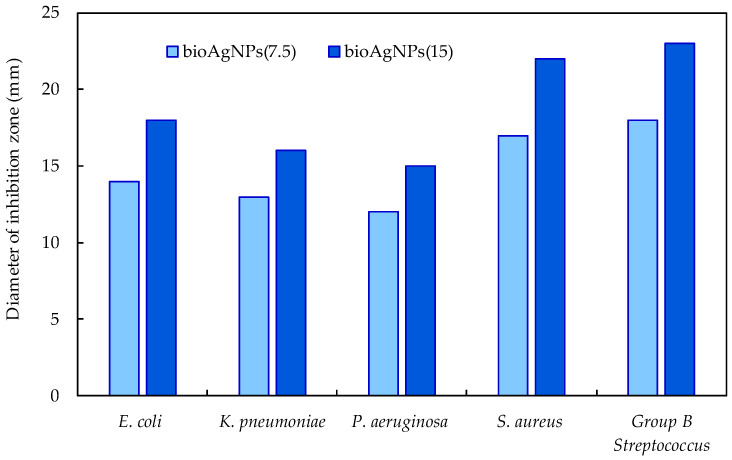
Dependence of the antibacterial activity of *bio*AgNPs.

Although *EchP* contains bioactive compounds (caffeic acid derivatives, alkamides, polysaccharides [33]), but in vitro studies of its aqueous extracts have shown only a weak antibacterial effect with the inhibition zone diameter not exceeding 11 mm. However, *bio*AgNPs synthesized using *EchP* aqueous extracts demonstrate markedly enhanced inhibitory activity. Figure 7 illustrates the concentration-dependent antibacterial activity of two colloidal solutions of *bio*AgNPs against Gram-positive and Gram-negative bacterial species. As expected, *bio*AgNPs(15) have stronger antibacterial effects, with inhibition zone diameters about 25–30% larger compared to *bio*AgNPs(7.5), highlighting the synergistic role of plant-derived compounds in nanoparticle synthesis and underscoring their potential for use in advanced antimicrobial formulations. Gram-negative bacteria exhibit slightly lower susceptibility to inhibition than Gram-positive bacteria, primarily due to their more resistant outer membrane.

Among the tested bacterial species, Group B *Streptococcus* is the most sensitive to inhibition (∅ 18–23 mm), while *P. aeruginosa* shows the smallest inhibition zone (∅ 12–15 mm). The antibacterial activity of *bio*AgNPs may be attributed to several mechanisms, including bacterial membrane disruption, DNA inactivation, protein denaturation, ribosome degradation, and adenosine triphosphate molecules damage [47,48]. In most cases, *bio*AgNP activity is strongly influenced by particle size, pH of the medium, the nature of capping agents, etc. On the other hand, the antibacterial efficiency of herbal extracts and biosynthesized nanoparticles may vary depending on the origin of plant material, its geographical source, cultivation conditions, extraction methods, and other variables. Consequently, results reported in different studies may differ [25]. However, many researchers agree that although plant-derived compounds have antimicrobial potential, their effectiveness is often limited [49,50,51]. Therefore, plant-derived compounds are most commonly used as supplementary rather than primary antimicrobial agents. In contrast, AgNPs synthesized with plant extracts typically produce much larger inhibition zones (often 15–20 mm or more), confirming the synergistic effect between silver and plant phytochemicals [52].

### 2.3. Properties of and Structure of KerP Electrospun Mats

The influence of the electrospun KerP solution composition on viscosity and conductivity is listed in Table 6. The addition of 7.5% and 15% aqueous extract of *EchP* leads to a significant reduction in the solution viscosity—by approx. 25% and 45%, respectively, due to the dilution effect and the presence of low-molecular-weight phytochemicals that disrupt polymer chain entanglement [53]. In contrast, incorporating equivalent amounts of the bioAgNP solution results in a comparatively smaller decrease in viscosity, by about 20% and 40%, respectively. This difference may be attributed to interactions between *bio*AgNPs and keratin molecules, potentially affecting the morphology of the electrospun composite. The conductivity of the spinning solution remains largely unaffected by the *EchP* extract content. However, the presence of *bio*AgNPs leads to a slight increase in conductivity, ranging from approximately 2% to 4%, likely due to nanoparticle-induced ionic contributions.

The FTIR spectra of the KerP electrospun mat exhibit characteristic absorption bands corresponding to peptide linkages (–CONH–), as previously illustrated in Figure 2. Changes in other regions of the KerP are summarized in Table 7. A distinct absorption peak at 2882 cm^−1^ is attributed to the symmetric and asymmetric stretching vibrations of methylene (CH_2_) groups within the PEO backbone. The presence of the crystalline phase of PEO is confirmed by a triplet of C–O–C stretching vibrations observed at 1140, 1099, and 1059 cm^−1^ [54]. Two PEO crystalline conformations—planar and helical—are observed in KerP spectra. The characteristic bands of the planar structure appear at 1340 (–CH_2_– wagging), 1240 (–CH_2_– twisting), and 961 cm^−1^ (–CH_2_– rocking), whereas the bands at 1359 (–CH_2_– wagging), 1278 (–CH_2_– twisting), 945, and 841 cm^−1^ (–CH_2_– rocking) are characteristic for the helical conformation of PEO [55]. Incorporation of *EchP* extract does not significantly alter the chemical structure of the KerP micro/nanofibers. However, the presence of plant-derived compounds induces a slight redshift in the broad band associated with O–H and N–H stretching, accompanied by an increase in intensity, suggesting enhanced hydrogen bonding [56]. Moreover, amide I and II bands exhibit shifts, indicating interactions between keratin and phytochemicals such as flavonoids and phenolics, likely mediated through hydrogen bonding. In the KerP/*bio*AgNP structure, further shifts in the amide I and II bands are observed, which are consistent with coordination interactions between protein functional groups and Ag^+^ or AgNPs, which suggest the formation of Ag–N or Ag–O bonds [57].

**Figure 8:**
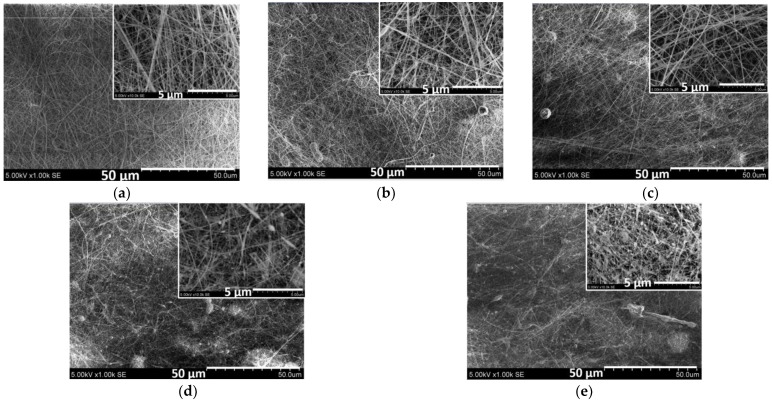
SEM micrographs of electrospun wool KerP nano-microfibers with varying compositions: (**a**) KerP (control), (**b**) KerP/*EchP*(7.5), (**c**) KerP/*EchP*(15), (**d**) KerP/*bio*AgNPs(7.5), (**e**) KerP/*bio*AgNPs(15).

**Figure 9:**
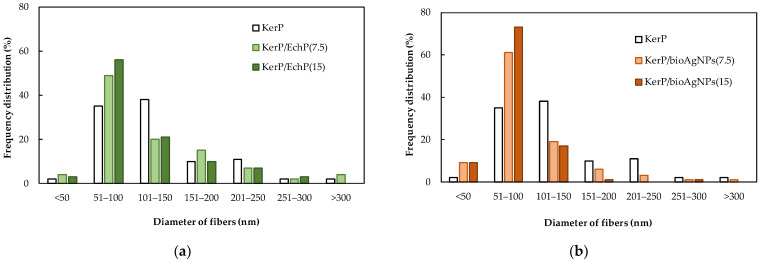
Distribution of the keratin nano-microfibers diameter from KerP/*EchP* blend (**a**) and KerP/*bio*AgNP blend (**b**).

The SEM micrographs in Figure 8 provide an insight into the composition-dependent changes in the morphology of the electrospun KerP micro/nanofibres. It is evident that the surface of the micro/nanofibers is smooth, free of bead defects, exhibiting features typical of porous and fibrous structures (Figure 8a). The diameter of pure KerP fibres predominantly ranged from 70 to 150 nm, with an average diameter of approximately 132 ± 64 nm (Figure 9a). The addition of *EchP*(7.5) and *EchP*(15) decreased the average fiber diameter to 120 ± 66 nm and 108 ± 54 nm, respectively. However, the incorporation of *EchP* extract into the spinning solution results in the formation of some droplets and bead-like defects, likely due to a reduction in the viscosity of the solution, which compromises the stability of the electrospinning jet, resulting in incomplete fiber formation and the formation of beads (Figure 8b,c).

Similarly, the addition of *bio*AgNPs(7.5) and *bio*AgNPs(15) in KerP solution decreases average fibre diameter from ~132 nm to 100 ± 58 nm and 82 ± 31 nm, respectively (Figure 9b). This reduction is primarily attributed to a decrease in solution viscosity and a slight increase in electrical conductivity. Lower viscosity facilitates the stretching of the polymer jet during electrospinning, while increased conductivity enhances the elongation forces acting on the jet, both contributing to the formation of finer fibres [58]. As the concentration of *bio*AgNPs increases, the frequency distribution of nanofibre diameters shifts toward smaller ranges, particularly within the 51–100 nm interval. This indicates not only a reduction in average diameter but also a narrowing of the diameter distribution, suggesting more uniform fibre formation in the case of *bio*AgNPs(15). However, an increase in *bio*AgNP concentration adversely affects the morphological integrity of the micro/nanofibers. The SEM micrographs reveal a pronounced increase in the number of droplets and bead-like defects (Figure 8d,e). Similarly, Ref. [58] reported that higher AgNP concentrations in electrospun fibers resulted in finer and more uniform fibers, but also led to an increased occurrence of bead-like defects. Additionally, the presence of bioactive *EchP* and *bio*AgNPs may interfere with the KerP polymer chain entanglement, further contributing to defect formation.

A decrease in solution viscosity was associated with an increase in porosity and changes in pore size of the electrospun mats. The KerP solution exhibited the highest viscosity (see Table 6), resulting in mats with the lowest porosity (~46%) but a relatively large average pore size (~180 nm) (Figure S2 in Supplementary File). The incorporation of *EchP* or *bio*AgNPs(7.5) reduced the viscosity, which facilitated enhanced jet elongation during electrospinning. This led to mats with 20–30% higher porosity and a decreased pore size with approximately 100–110 nm. The findings observed between viscosity and porosity in KerP-based electrospun mats have been reported by other researchers. Chen et al. demonstrated that higher viscosity of polycaprolactone solutions contributes to higher fiber diameters and increased pore sizes in electrospun microtubes [59]. However, in the case of SEM images of KerP+*bio*AgNPs (15), which revealed the presence of droplets and bead-like defects, porosity and pore size values were similar to those observed for KerP mats. It can be proposed that these defects alter the surface appearance, and therefore the porosity determined by ImageJ analysis (Version 1.53; National Institutes of Health, USA) appears to decrease.

**Figure 10:**
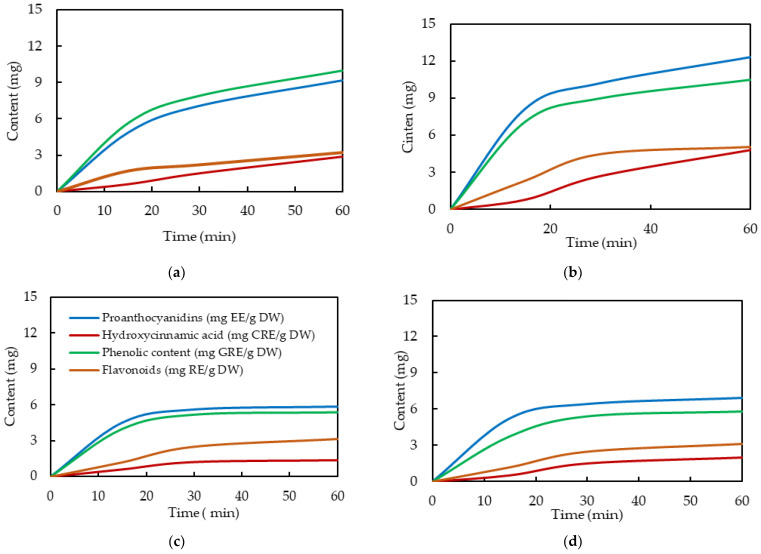
Active compound release kinetics for electrospun keratin mats: KerP/*EchP*(7.5) (**a**), KerP/*EchP*(15) (**b**), KerP/*bio*AgNPs(7.5) (**c**), KerP/*bio*AgNPs(15) (**d**).

Short-term release kinetics were studied to elucidate the role of plant-derived bioactive compounds in the capping and stabilization of green-synthesized *bio*AgNPs. The results, summarized in Figure 10, reveal differences in the release rate and mechanism depending on the type and concentration of additives incorporated into the KerP matrix.

The release of tannins (proanthocyanidins) and phenolic compounds is more than 2–3 times higher than that of the hydroxycinnamic acid derivatives and flavonoids. As the concentration of the *EchP* in the KerP mats increases, the content of compounds released also increases (in 1–3 mg). As shown in Figure 10c,d, the intensity of release of bioactive compounds from KerP/*bio*AgNP mats is markedly lower compared to that of KerP/*EchP*, decreasing by approximately 1.3–1.6 times. This supports the assumption that secondary metabolites are not only involved in the bioreduction of AgNPs, but also act as capping and stabilising agents to prevent their aggregation [36,60]. Their interaction with the nanoparticle surface likely reduces their mobility and availability for release, resulting in a slower and more sustained release profile. The data suggest that while KerP/*EchP*-based mats offer rapid and high-intensity release of bioactive additives, KerP/*bio*AgNP-based mats provide a more controlled and sustained release. This confirms the role of polyphenols as electron donors, facilitating the bioreduction of Ag^+^ to Ag^0^ and contributing to the stabilization of nanoparticles.

Overall, the release profiles of active compounds from electrospun keratin mats show an initial rapid release followed by a plateau phase, which is characteristic of diffusion-controlled systems. A theoretical comparison suggests that the observed release behavior does not align with the zero-order kinetic model, which assumes a constant release rate over time and would produce a linear increase in concentration. Similarly, the data do not follow the first-order kinetic model, which typically represents an exponential decrease in release rate over time. The release pattern appears more consistent with the Higuchi model, which describes drug release as a diffusion process based on the square root of time. The initial burst followed by a slower release phase suggests that diffusion from the polymer matrix may be the dominant mechanism, particularly in the early stages. Additionally, the Korsmeyer–Peppas kinetic model may be applicable, as it is often used to describe drug release from polymeric systems where multiple mechanisms may be involved. These theoretical observations are consistent with previous findings reported by Gencturk et al. [61], where the Higuchi and Korsmeyer–Peppas models were found to best describe diffusion-controlled release from electrospun nanofibers.

## 3. Materials and Methods

### 3.1. Materials

Wool was purchased from Romanian sheep farmers. Chemical reagents, such as sodium hydroxide (NaOH), ammonia (25%), sodium carbonate (Na_2_CO_3_), formic acid (HCOOH), purchased from Chimopar SA (Bucharest, Romania), and silver nitrate (AgNO_3_, ≥99.8%), polyethylene oxide (PEO, Mw~100,000), obtained from Sigma Aldrich (USA), were of analytical grade. Borron SE (ethoxylated alkyl derivatives with 65% concentration) was supplied by SC Triderma SRL (Bucharest, Romania). The crushed *EchP* flower blossoms were obtained from Švenčionių vaistažolės UAB (Švenčionys, Lithuania).

### 3.2. Methods

#### 3.2.1. Keratin Hydrolysate Preparation and Characterization

**Figure 11:**
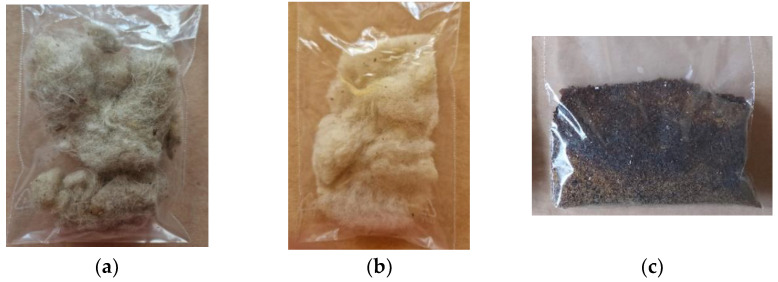
Examples of raw material: sheep raw wool (**a**), degreased and washed wool (**b**), keratin hydrolysate (**c**).

The keratin hydrolysate was prepared from raw wool of coarse quality (Figure 11a), which was collected from sheep breeders, according to the methods described in [62,63,64]. The wool was degreased and washed in a compact aqueous process using 4% *v*/*v* ammonia, 1% *w*/*v* sodium carbonate, and 0.6% *w*/*v* Borron SE degreasing agent, at 40 °C for 2 h. After that the washing water was drained and the wool was washed at least three times with cold water until a neutral pH was reached. The washed wool (Figure 11b) was squeezed, dried, and cut with a bench grinding machine (La Minerva, Minerva Omega, Bologna, Italy) and hydrolyzed with 2.5% *w*/*v* sodium hydroxide at 80 °C for 4 h. The wool was almost completely solubilized (96.5%) and was decanted, filtered, and dried in an oven with convective air at 60 °C and the solid keratin was ground using a mortar or a ball mill (Figure 11c).

Obtained wool keratin was characterized according to valid standards or literature methods: volatile matter—ISO 4684; total ash content—ISO 4047; nitrogen and protein contents—ISO 5397; electrical conductivity—EN 2788; and pH of 10% w/v solution—STAS 8619/3. The protein content was calculated using 6.06 conversion factor according to the method presented in [65].

The molecular weight was determined by using Agilent 1260 GPC system (Agilent Technologies, Santa Clara, CA, USA) equipped with a PL aqua gel-OH MIXED-H column (7.5 × 300 mm, 8 µm) and a multi-detection unit. Optimum working conditions were chosen flow rate of mobile phase containing 1 mL·min^−1^, the injection volume of the sample was 100 µL, and the temperature of 35 °C for the detectors and column. SDS-PAGE electrophoresis was also performed according to the Laemmli method [66] using a Mini Protean 3 Cell (Mini PROTEAN 3 Cell Bio-Rad, Hercules, CA, USA). The proteins were separated in a 12.5% gel run at 30 V for 30 min and at 100 V for 120 min. A molecular weight marker ranging from 10 to 250 kDa (Bio-Rad) was used. The gels were stained with 0.2% Coomassie Brilliant Blue R-250 solution. The lanes and band ratios were measured with Gel Doc EZ Imaging System and analyzed with ImageLab software, version No. 6.0.1.

The average particle size, polydispersity and Zeta potential characteristics were measured using a Zetasizer Nano-ZS device (Malvern Instruments, Malvern, UK) on keratin dispersion of 0.3% *w*/*v* concentration, at 25 °C, in triplicate. The viscosity was measured by using a Brookfield DV2T Viscometer (Toronto, ON, Canada), and the electrical conductivity with an Orion Star A211 Benchtop potentiometer (Thermo Fisher Scientific Inc., Waltham, MA, USA). The proteinogenic amino acid analyses were performed with the help of HPLC-Amino Acid Analyser LC3000 (Sykam GmbH, Eresig, Germany), equipped with a polymeric cation exchanger column, post-column ninhydrin derivatization at 125 °C, and photometric measurement at 570 nm. The results were monitored using ChromStar chromatography software (version 6.0), and reported as a mean of triplicate determinations.

FTIR measurements for keratin hydrolysate were performed on keratin film using an INTERSPEC 200-X spectrophotometer (Interspectrum, Tartumaa, Estonia) equipped with an ATR device. The spectra of the samples were achieved in triplicate by examining the frequency ranging from 4000 cm^−1^ to 700 cm^−1^ with a 2 cm^−1^ resolution and placing the keratin hydrolysate powder on a ZnSe crystal. Amide I band deconvolution was performed to identify the ordered structures affected by alkaline hydrolysis and to correlate with the bioactive properties. Thus, β-sheets, random coils, and α-helix were examined in the ranges of 1613 cm^−1^–1637 cm^−1^, 1637 cm^−1^–1645 cm^−1^, and 1645 cm^−1^–1662 cm^−1^, respectively [67]. The deconvolution parameters were settled in the spectra range of 1700–1600 cm^−1^ using a Lorentzian function. Secondary structure of wool keratin hydrolysate was obtained by using the ratio between the individual area band to the whole area bands.

#### 3.2.2. Green Synthesis of Silver Nanoparticles (*bio*AgNPs) and Characterization

Silver nanoparticles can be successfully synthesized using biologically active functional groups and interaction sites present in medical plant extracts, which acts as a reducing, stabilizing and capping agent [36]. In this study, an aqueous extract of *EchP* was used. Dried flowers were first ground into a fine powder using a laboratory mill (IKA^®^ A11 basic, Staufen, Germany) (Figure 3a–c). Moisture content was determined by drying approximately 1 g of the powdered material in a moisture analyzer (Precisa HA 300, Precisa Instruments AG, Dietikon, Switzerland) at 103 °C until complete evaporation of water and volatile compounds. The results were recalculated to express the absolute dry weight (DW), which was approximately 10 mg/mL. For extract preparation, 1 g of the powdered plant material was mixed with 100 mL of distilled water and heated at 90 °C under constant stirring for 15 min. The resulting extract was filtered and stored at 4–6 °C until use in nanoparticle synthesis. To synthesize *bio*AgNPs, 30 mg of AgNO_3_ (corresponding to approximately 5 mM) was dissolved in 2.5 mL of ultrapure water and mixed with 30 mL of the *EchP* aqueous extract under vigorous stirring at room temperature for 2 h. The mixture was then incubated at room temperature for 24 h to allow the formation of a stable *bio*AgNP colloidal solution (Figure 3d). The final product, with a nanoparticle concentration of approx. 0.58 mg/mL, was stored in a cool, dark environment to preserve its stability and bioactivity.

The formation of *bio*AgNPs was evidenced by a distinct color change in the colloidal solution. Quantitative colorimetric analysis was conducted using a MiniScan XE Plus spectrophotometer (Hunter Associates Laboratory, Inc., Reston, Virginia, USA), which operates within the CIELAB color space. It is often used to quantify colored chemicals due to its perceptual uniformity and ability to represent subtle color differences [68]. Color measurements were expressed in terms of three coordinates: L* (lightness), representing the luminance component of a colour, a* (green-red axis) and b* (blue-yellow axis), representing colour position. From these primary coordinates, two derived parameters were calculated: chroma (C*), representing color saturation (C* = (a* × 2 + b* × 2)^1/2^) and hue angle (*h*), representing the color tone (*h* = arctan(b*/a*)). Measurements were recorded at 15 min intervals until stabilization of the colorimetric parameters was observed, indicating the completion of nanoparticle synthesis.

The size and morphology of the biosynthesized silver nanoparticles (*bio*AgNPs) were evaluated using a transmission electron microscope (TEM, Tecnai G2 F20 X-TWIN, FEI, Hillsboro, OR, USA) with a resolution range of 0.8–1.0 nm. A diluted *bio*AgNP solution was deposited dropwise onto carbon-coated copper TEM grids for imaging. The instrument was equipped with a Schottky field emission electron source and operated at an accelerating voltage ranging from 20 to 200 kV. The crystalline structure of the synthesized *bio*AgNPs was confirmed by X-ray diffraction (XRD) using a Bruker D8 diffractometer (Bruker, MA, USA) with Cu Kα radiation (λ = 0.15418 nm). Scans were performed over a 2θ range of 20–70° with a step size of 0.01 degree.

Fourier-transform infrared spectroscopy (FT-IR) was used to analyze the chemical structure of the EchP extract and the *bio*AgNPs. Reflectance spectra were recorded using a Vertex 70 spectrophotometer (Bruker, MA, USA). A small amount of each sample was pressed against a crystal plate, and spectra were collected over the range of 4000–650 cm^−1^ with a resolution of 4 cm^−1^ and 16 scans per sample. Data was processed using OPUS 7.8 software (Bruker Optics, Billerica, MA, USA). For FT-IR analysis, thin films of *EchP* and *bio*AgNPs were deposited on silicon substrates.

#### 3.2.3. Keratin/PEO Electrospinning Solutions Preparation and Characterization

The brittle behavior and weak mechanical properties of wool keratin hydrolysate restrict its use in practical applications [5]. To improve its spinnability and mechanical performance, it was blended with high-molecular-weight poly(ethylene oxide) (PEO)—an amphiphilic, water-soluble, and biocompatible polymer widely used in biomedical applications [13]. For the preparation of the KerP solution, 90 g of keratin hydrolysate was dissolved in 90 g of distilled water and stirred for 16 h at 25 °C. This solution was then mixed with 450 g of a 10% (*w*/*w*) aqueous PEO solution and stirred for an additional 8 h at the same temperature. Both the dissolution and mixing processes were carried out using a shaker (KS 4000, IKA, Staufen im Breisgau, Deutschland) at a speed of 150 rpm. The detailed methodology for preparing the KerP solution is described in Ref. [69].

Prior to the electrospinning process, the extract of *EchP* or the *bio*AgNP colloidal solution was added dropwise to the KerP solution. The compositions of the KerP formulations used in this study are summarized in Table 8.

The electrical conductivity of electrospinning solutions was measured using a portable multi-meter HQ40d (HACH, Loveland, CO, USA). Measurements were performed in triplicate at room temperature, and the average value was recorded as the final result. Viscosity measurements were conducted at room temperature using a rotational viscometer (Fungilab Smart Series, Barcelona, Spain) operating at a spindle speed of 50 rpm. Each sample was measured three times, and the average value was taken as the representative viscosity.

#### 3.2.4. Keratin Mats Electrospinning and Characterization

Nano-microfibers were fabricated using Nanospider™ electrospinning equipment (Elmarco, Liberec, Czech Republic) under the following conditions: applied voltage U = 65 kV, electrode distance L = 13 cm, ambient temperature T = 20 ± 2 °C, and relative humidity RH = 58 ± 5%. The fiber collection time on the polypropylene spunbond support material was 60 min. The morphology of the electrospun mats was examined using a scanning electron microscope (SEM S-3400N, Hitachi, Japan). The average diameter of the electrospun nano-microfibers was determined from 100 measurements taken from SEM images at 10,000× magnification (scale bar 5 µm), using NIS-Elements D software, Version 5.02 (Nikon Corporation, Japan).

#### 3.2.5. Bioactivity Assay

Quantitative phytochemical analysis of the *EchP* extract and the *bio*AgNP colloidal solution was performed using a double-beam UV-Vis scanning spectrophotometer (M550, Spectronic CamSpec, Garforth, UK). Several analytical methods were employed to determine the content of active compounds in the extract: TPC was measured using the Folin–Ciocalteu assay; TFC was determined using the aluminum chloride colorimetric method; total proanthocyanidin content was assessed using the DMCA assay; and total hydroxycinnamic acid content was quantified by a reagent-based colorimetric assay.

The antibacterial activity of the *EchP* extract and the *bio*AgNPs was evaluated in vitro using the Agar diffusion test [70]. Sterile cellulose discs (area ≈ 1 cm^2^) were impregnated with *EchP* aqueous extract or with *bio*AgNP colloidal solutions (fixed volume per disc, 20–30 µL), then air-dried aseptically until solvent had evaporated. Gram-positive bacterial strains (*Staphylococcus aureus* ATCC 25923 (*S. aureus*), *Streptococcus agalactiae* ATCC 19563 (Group B Streptococcus, GBS) and Gram-negative bacteria strains (*Klebsiella pneumoniae* ATCC 13883 (*K. pneumoniae*), *Escherichia coli* ATCC 25922 (*E. coli*) and *Pseudomonas aeruginosa* ATCC 27853 (*P. aeruginosa*)) were selected for antimicrobial testing. Standardized bacterial suspensions adjusted to 0.5 McFarland (~1 × 10^8^ CFU/mL) were inoculated onto cooled Mueller–Hinton agar plates (Oxoid, Basingstoke, UK) using sterile cotton swabs. The prepared discs were placed on the inoculated plates and incubated at 37 °C for 24 h. Zones of growth inhibition were then measured in millimeters for each bacterial strain.

For the release study of phenolics, flavonoids, proanthocyanidins, and hydroxycinnamic acids, 200 mg of the keratin mat was immersed in 50 mL of infusion medium (Ringer B. Braun saline solution, 500 mL, N10) and stirred using a magnetic stirrer at room temperature for 24 h. After a fixed period of time, the mat was removed, and the concentration of released phytochemicals in the solution was analyzed using the same methods described above.

### 3.3. Statistical Analysis

All experiments were conducted in triplicate. The mean values and standard deviations were calculated using STATISTICA 10 software (StatSoft, Inc., Tulsa, OK, USA) and Microsoft Excel (Microsoft Corp., Redmond, WA, USA). Statistical analysis was performed using one-way analysis of variance (ANOVA), followed by Tukey’s Honestly Significant Difference (HSD) post hoc test. Differences were considered statistically significant at *p* < 0.05.

## 4. Conclusions

In this study, keratin-based electrospun mats were successfully fabricated by incorporating *Echinacea purpurea* L. (*EchP*) extract and biosynthesized silver nanoparticles (*bio*AgNPs) using this extract. The silver nanoparticles, with an average diameter of 20.5 nm, were well-dispersed and exhibited minimal agglomeration due to effective stabilization by phytochemicals present in *EchP* extract. While aqueous extracts of *EchP* exhibit only weak antibacterial activity, *bio*AgNPs show concentration-dependent inhibition of both Gram-positive and Gram-negative bacterial strains. At double the concentration, *bio*AgNPs produced inhibition zones approximately 25–30% larger.

The electrospinning of keratin resulted predominantly in micro/nanofibers with diameters below 100–150 nm. However, the addition of *EchP* extract or *bio*AgNPs led to the formation of bead-like structures within the fiber matrix, likely due to a reduction in solution viscosity. On the other hand, the change in rheological properties contributed to the formation of finer and more uniform diameter fibers.

The green synthesis of *bio*AgNPs using *EchP* extract reduced the release of bioactive compounds from the electrospun mats, suggesting that these compounds act as electron donors in Ag^+^ bioreduction and contribute to nanoparticle stabilization.

## Figures and Tables

**Table 1:** Main wool keratin hydrolysate characteristics.

Characteristic	Value
Volatile matter (%)	9.00 ± 0.35
Total ash (%)	14.40 ± 0.24
Total nitrogen (%)	12.50 ± 0.34
Protein substance (%)	75.75 ± 0.34
Aminic nitrogen (%)	0.75 ± 0.05
Molecular weight (Da)	11,315
pH (pH units)	10.30 ± 0.10
Electrical conductivity (μS/cm)	13,700
Viscosity (cP)	47
Average particle size (nm)	2353
Polydispersity (a.u.)	0.42
Zeta potential (mV)	−11.4
Proteinogenic amino acids (% of protein):	
glutamic acid	17.89 ± 0.15
aspartic acid	13.60 ± 0.12
leucine	9.88 ± 0.08
proline	7.64 ± 0.70
arginine	7.46 ± 0.06
valine	7.03 ± 0.06
glycine	6.52 ± 0.06
alanine	5.40 ± 0.05
serine	5.03 ± 0.04
tyrosine	5.03 ± 0.04
isoleucine	4.47 ± 0.04
phenylalanine	4.28 ± 0.04
threonine	2.24 ± 0.02
lysine	2.42 ± 0.02
cysteine	1.86 ± 0.02
histidine	0.80 ± 0.01

**Table 2:** Molecular weight analysis data of keratin hydrolysate.

Band No.	Mw (Da)	Relative Front	Adj. Volume (Int)	Volume (Int)	Band (%)	Lane (%)
1	250,000	0.043	33,698	3,285,033	6.7	6.7
2	250,000	0.092	20,851	2,766,977	4.1	4.1
3	14,000	0.771	451,675	29,961,669	89.2	89.2
4	10,000	0.863	232	2,340,039	0.0	0.0

**Table 3:** Amide I band deconvolution in the range of 1700–1600 cm^−1^ evaluated for keratin sample as compared to wool.

Material	β-Sheets, %	Random Coils, %	α-Helix, %	Turns, %
Wool keratin hydrolysate	26.88	53.55	19.56	–
Wool [32]	10.00	–	56.00	25

**Table 4:** Changes in the colorimetric parameters of *EchP* extract, indicating the formation of *bio*AgNPs.

Time, min	L*	a*	b*	C*	*h*
0	25.92 ± 1.05	7.06 ± 0.02	6.73 ± 0.04	9.76 ± 1.04	43.62 ± 1.02
15	26.07 ± 0.70	7.36 ± 1.01	5.91 ± 0.02	9.44 ± 0.20	38.75 ± 1.04
30	25.35 ± 0.71	6.93 ± 0.07	3.90 ± 0.07	7.95 ± 0.10	29.41 ± 2.14
45	25.22 ± 0.02	6.44 ± 0.25	3.86 ± 0.10	7.51 ± 0.02	30.92 ± 0.07
60	24.22 ± 0.31	4.16 ± 0.02	2.56 ± 0.05	4.88 ± 0.20	31.63 ± 0.30

**Table 5:** Phytochemical analysis of *EchP* aqueous extract and synthesized *bio*AgNP colloidal solution.

Compound Name	*EchP*	*bio*AgNPs
The total content of: proanthocyanidins, mg EE/g DW	24.56 ± 0.4	15.13 ± 0.24
hydroxycinnamic acid derivatives, mg CRE/g DW	17.30 ± 0.45	6.55 ± 0.24
phenolic compounds, mg GRE/g DW	74.23 ± 1.29	60.02 ± 1.78
flavonoids, mg RE/g DW	38.14 ± 0.67	21.50 ± 0.24

Note: mg EE/g DW is milligrams of proanthocyanidins per gram of dry weight (DW), expressed as Epicatechin Equivalents (EE); mg CRE/g DW is milligrams of hydroxycinnamic acid derivatives per gram of dry weight, expressed as Caffeic Acid Equivalents (CRE); mg GRE/g DW is milligrams of total phenolic compounds per gram of dry weight, expressed as Gallic Acid Equivalents (GRE); mg RE/g DW is milligrams of flavonoid content per gram of dry weight, expressed as Rutin Equivalents (RE).

**Table 6:** Dependence of the viscosity and conductivity of wool KerP aqueous solutions upon composition.

Composition	Viscosity, mPa·s		Conductivity, μS/cm	
	Average	±SD	Average	±SD
KerP	164.0	0.1	22.0	0.1
KerP/*EchP*(7.5)	123.3	0.6	22.1	0.1
KerP/*EchP*(15)	91.8	0.3	22.0	0.1
KerP/*bio*AgNPs(7.5)	133.3	0.1	22.8	0.1
KerP/*bio*AgNPs(15)	100.6	0.6	22.4	0.1

**Table 7:** Additional FTIR peak positions and changes for KerP composition.

Functional Group	Peak (cm^−1^):		
	KerP	KerP/*EchP*	KerP/*bio*AgNPs
O–H and N–H stretching	3245	3242	3240
Methylene (CH_2_) groups stretching (PEO)	2882	2882	2882
Amide I (C=O stretching)	1653	1645	1640
Amide II (N–H bending, C–N stretching)	1545	1540	1536
C–O–C stretching (PEO crystalline phase indicator) Planar conformation of PEO Helical conformation of PEO	1140, 1099, 1060 1340, 1240, 961 1359, 1278, 945, 841	1140, 1099, 1060 1340, 1240, 961 1359, 1278, 945, 841	1140, 1099, 1060 1340, 1240, 961 1359, 1278, 945, 841

**Table 8:** Keratin/PEO electrospinning solutions for electrospun nano-microfibre formation.

Code of Sample	Amount (%)				
	Keratin Hydrolysate	PEO Aqueous Solution (*c* = 10%)	Water	*EchP* Aqueous Extract (10 mg/mL)	*bio*AgNP Colloidal Solution (0.58 mg/mL)
KerP	14	72.0	14	–	–
KerP/*EchP*(7.5)	13	66.5	13	7.5	–
KerP/*EchP*(15)	12	61.0	12	15.0	–
KerP/*bio*AgNPs(7.5)	13	66.5	13	–	7.5
KerP/*bio*AgNPs(15)	12	61.0	12	–	15.0

## Data Availability

Data present in this study are available upon reasonable request from the corresponding author.

## Supplementary Materials

The following supporting information can be downloaded at: https://www.mdpi.com/article/10.3390/ijms26209919/s1.
