# Supplementary material for: Fabrication and Characterization of Electrospun Keratin Mats with Echinacea purpurea L. and Biosynthesized Silver Nanoparticles
Source: Int J Mol Sci. 2025 Oct 12;26(20):9919. doi: 10.3390/ijms26209919 (PMC12563298; doi:10.3390/ijms26209919)
Supplement: Supplementary file 1 [file ijms-26-09919-s001.zip › ijms-3873234-supplementary.pdf]

Supplementary Materials

# Fabrication and Characterization of Electrospun Keratin Mats with *Echinacea Purpurea* L. and Biosynthesized Silver Nanoparticles

**Akvilė Andziukevičiūtė-Jankūnienė, Erika Adomavičiūtė, Carmen Gaidau, Virgilijus Valeika, Aistė Balčiūnaitienė, Jonas Viškelis, Maria Rapa, and Virginija Jankauskaitė**

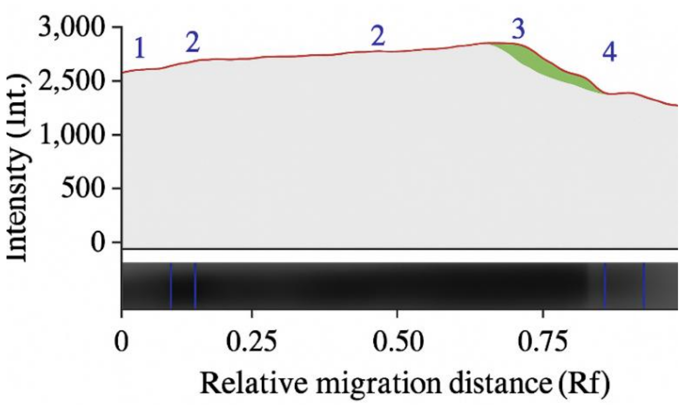


**Figure S1.** SDS-PAGE electrophoresis bands and lanes of keratin hydrolysate

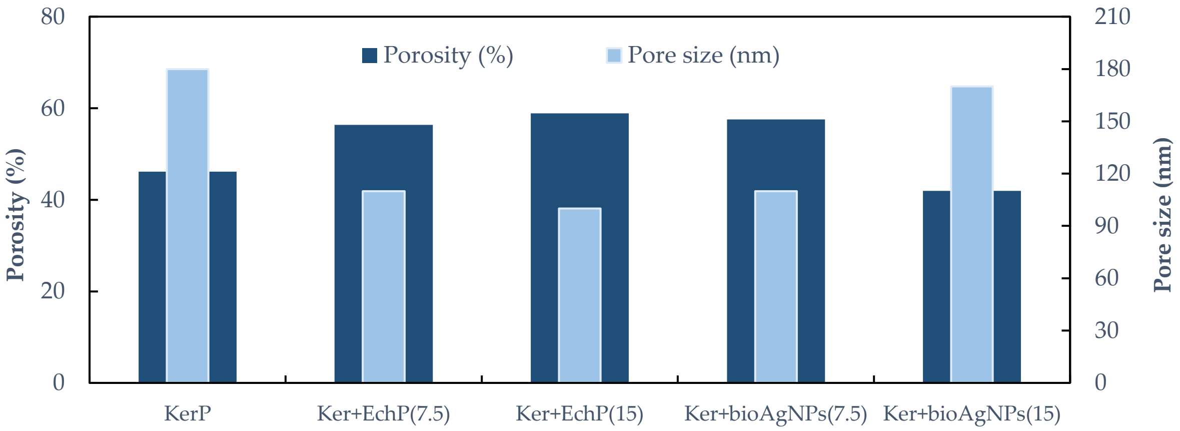


**Figure S2.** Dependence of average porosity and pore diameter on electrospun mats composition (obtained using *ImageJ* software from SEM images)

The porosity and average pore diameter of the samples were evaluated from SEM micrographs using the open-source software ImageJ/Fiji (version 1.53, National Institutes of Health, USA). SEM images were first

converted to 8-bit grayscale and then thresholded to distinguish pores from the solid matrix. The “Analyze Particles” function was used to quantify the pore area fraction (porosity) and to measure the equivalent pore diameters. The scale was calibrated using the scale bar provided in each SEM image. For each sample, at least three representative images were analyzed, and the results were averaged.

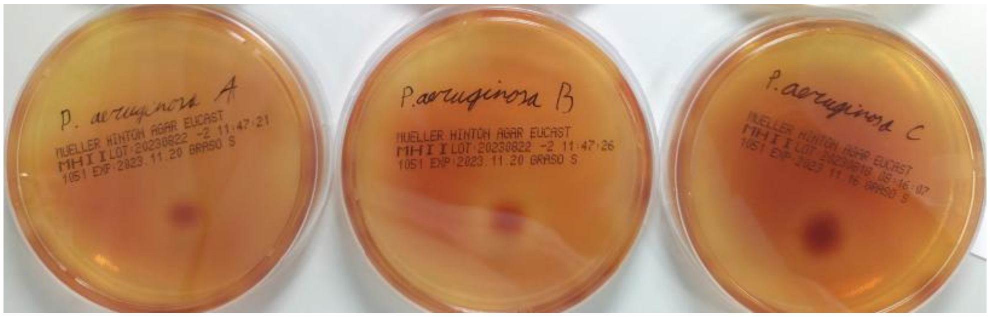


**Figure S3.** The antibacterial activity of KerP/*bio*AgNPs(7.5) mats against *P. aeruginosa*
